# Supplementary material for: Assembling metagenomes, one community at a time
Source: BMC Genomics. 2017 Jul 10;18:521. doi: 10.1186/s12864-017-3918-9 (PMC5502489; doi:10.1186/s12864-017-3918-9)
Supplement: Supplementary file 1 — Attributes of de novo assemblers used in this study. Included in this table are the versions of each assembler used in this study, along with the release date of each version. We provide a link to each assemblers’ website accompanied by its reference and number of citations. We gauge ease of use by providing the programming language and MPI compatibility of each tool as well as assessing the completeness of each tools’ available documentation. Table S2. Characteristics of the metagenomic datasets used in this study. Three metagenomes from three distinct environments (Soil, Aquatic and Human gut) were selected, and we provide accession numbers, sequencing platforms used and basic sequence characteristics (pre- and post-filtering) of each metagenome. Table S3. Assembly statistics for the assembled aquatic metagenomes. Table S4. Assembly statistics for the assembled soil metagenomes. Table S5. Assembly statistics for the assembled human gut metagenomes. Table S6. Assembly statistics for the synthetic metagenomes. Figure S1. Nonpareil estimates of sequence coverage (redundancy) for the 3 synthetic metagenomes studied. Figure S2. Computational requirements for the Tara Ocean metagenome. A) Total assembly span proportional to wall time required. B) Total assembly span in relation to peak memory usage. Figure S3. Correlation between assembly span and mapping rate. The exponential trendline indicates a very strong positive correlation between the amount of data utilized and the size of the generated assembly (R2 = 0.83). (DOCX 357 kb) [file 12864_2017_3918_MOESM1_ESM.docx]

**Additional files**

**Table S1.** **Attributes of *de novo* assemblers used in this study**. Included in this table are the versions of each assembler used in this study, along with the release date of each version. We provide a link to each assemblers’ website accompanied by its reference and number of citations. We gauge ease of use by providing the programming language and MPI compatibility of each tool as well as assessing the completeness of each tools’ available documentation.

**Table S2.** **Characteristics of the metagenomic datasets used in this study.** Three metagenomes from three distinct environments (Soil, Aquatic and Human gut) were selected, and we provide accession numbers, sequencing platforms used and basic sequence characteristics (pre- and post-filtering) of each metagenome.

**Table S3. Assembly statistics for the assembled aquatic metagenomes.**

**Table S4.** **Assembly statistics for the assembled soil metagenomes.**

**Table S5. Assembly statistics for the assembled human gut metagenomes.**

**Table S6. Assembly statistics for the assembled synthetic metagenomes.**

**Figure S1.** **Nonpareil estimates of sequence coverage (redundancy) for the 3 synthetic metagenomes studied.**

**Figure S2. Computational requirements for the Tara Ocean metagenome.** A) Total assembly span proportional to wall time required. B) Total assembly span in relation to peak memory usage.

**Figure S3. Correlation between assembly span and mapping rate**. The exponential trendline indicates a very strong positive correlation between the amount of data utilized and the size of the generated assembly (R^2^ = 0.83).

**Table S1.** **Attributes of *de novo* assemblers used in this study**. Included in this table are the versions of each assembler used in this study, along with the release date of each version. We provide a link to each assemblers’ website accompanied by its reference and number of citations. We gauge ease of use by providing the programming language and MPI compatibility of each tool as well as assessing the completeness of each tools’ available documentation.

| **Assembler** | **Version** | **Release Date** | **Language** | **MPI Compatibility** | **Reference** | **Citations** | **URL** |
| --- | --- | --- | --- | --- | --- | --- | --- |
| CLC | v.8.5.1 | October 2015 | Java | No |  | - | <https://www.qiagenbioinformatics.com/products/clc-genomics-workbench/> |
| IDBA-UD | v.1.1.2 | Jun 2014 | C++ | No | ^1^ | 627 | <http://i.cs.hku.hk/~alse/hkubrg/projects/idba_ud/> |
| MEGAHIT | v.1.0.6 | October 2016 | CUDA | Yes | ^2^ | 114 | <https://github.com/voutcn/megahit> |
| metaSPAdes | v.3.9.0 | July 2016 | Python | No | ^3^ | 18 | <http://cab.spbu.ru/software/spades/> |
| MetaVelvet | v.1.2.02 | September 2012 | C | Yes | ^4^ | 339 | <http://metavelvet.dna.bio.keio.ac.jp/> |
| Omega | v.1.4 | August 2016 | C++ | Yes | ^5^ | 29 | <http://omega.omicsbio.org/> |
| Ray Meta | v.2.3.1 | February 2014 | C++ | Yes | ^6^ | 245 | <http://denovoassembler.sourceforge.net/> |
| SPAdes | v.3.9.0 | July 2016 | Python | No | ^7^ | 1994 | <http://cab.spbu.ru/software/spades/> |
| Velvet | v.1.2.10 | April 2011 | C | Yes | ^8^ | 5974 | <https://www.ebi.ac.uk/~zerbino/velvet/> |

**Table S2.** **Characteristics of the metagenomic datasets used in this study.** Three metagenomes from three distinct environments (Soil, Aquatic and Human gut) were selected, and we provide accession numbers, sequencing platforms used and basic sequence characteristics (pre- and post-filtering) of each metagenome.

| **System** | **Metagenome** | **Accession No.** | **Reference** | **Platform** | **No. reads pre-QC** | **Mean read length (bp)** | **No. reads post-QC** |
| --- | --- | --- | --- | --- | --- | --- | --- |
| Soil | Iowa | SRR351474 | ^9^ | HiSeq 2000 | 822,282,876 | 100 | 470,688,764 |
|  | Permafrost | 4470009.3 - 4470010.3 | ^10^ | Genome Analyzer II | 35,337,078 | 114 | 25,860,604 |
|  | Oklahoma | SRR958082 | ^11^ | HiSeq 2000 | 113,445,350 | 101 | 91,465,330 |
| Aquatic | Kolkata Lake | 4673644.3 - 4673645.3 | Unpublished data | MiSeq | 55,969,488 | 151 | 36,376,806 |
|  | Tara Ocean | ERR598950 | ^12^ | HiSeq 2000 | 62,109,422 | 100,01 | 54,081,014 |
|  | Arctic Frost Flower | 4537104.3 - 4537105.3 | ^13^ | HiSeq 2000 | 42,024,360 | 101 | 37,690,670 |
| Human gut | Infant Gut | ERR526087 | ^14^ | HiSeq 2000 | 47,653,548 | 100 | 46,840,928 |
|  | European Gut | SRR341725 | ^15^ | HiSeq 2000 | 35,680,006 | 101 | 23,069,870 |
|  | Scandinavian Gut | ERR732914 | ^16^ | HiSeq 2000 | 26,730,558 | 101 | 21,820,314 |
| Simulated | Low Complexity | RL_S001__insert_270 | https://data.cami-challenge.org/ | Simulated | 100,000,000 | 150 | 99,771,962 |
|  | Mid Complexity | RM2_S001__insert_270 | https://data.cami-challenge.org/ | Simulated | 100,000,000 | 150 | 99,797,512 |
|  | High Complexity | RH_S001__insert_270 | https://data.cami-challenge.org/ | Simulated | 100,000,000 | 150 | 99,748,632 |

**Table S3.** **Assembly statistics for the assembled soil metagenomes.**

|  | **Permafrost** | | | | | | | | | |
| --- | --- | --- | --- | --- | --- | --- | --- | --- | --- | --- |
|  | **CLC** | **IDBA-UD** | **MEGAHIT** | **metaSPAdes** | **MetaVelvet** | **Omega** | **Ray Meta** | **SPAdes** | **Velvet** |  |
| Number of contigs (≥ 500 bp) | 7,471 | 37,100 | 42,893 | 27,964 | 2,512 | 319 | 612 | 45,825 | 2,512 |  |
| Total length | 6,368,740 | 35,345,296 | 38,460,071 | 26,197,278 | 1,861,803 | 510,449 | 563,997 | 47,250,172 | 1,861,803 |  |
| No. of long contigs (≥ 1 kbp) | 1,484 | 8,992 | 8,732 | 6,080 | 315 | 315 | 143 | 12,488 | 315 |  |
| No. of ultra-long contigs (≥ 50 kbp) | 0 | 0 | 0 | 1 | 0 | 0 | 0 | 4 | 0 |  |
| Largest contig | 12,914 | 35,547 | 46,328 | 80,920 | 3,975 | 16,034 | 14,465 | 94,431 | 3,975 |  |
| *N50* | 816 | 949 | 862 | 910 | 716 | 1,544 | 898 | 1,060 | 716 |  |
| *L50* | 2,243 | 10,020 | 12,224 | 7,460 | 917 | 101 | 174 | 11,134 | 917 |  |
|  | | | | | | | | | | |
|  | **Oklahoma** | | | | | | | | | |
|  | **CLC** | **IDBA-UD** | **MEGAHIT** | **metaSPAdes** | **MetaVelvet** | **Omega** | **Ray Meta** | **SPAdes** | **Velvet** | |
| Number of contigs (≥ 500 bp) | 1,151 | 21,577 | 27,650 | 17,886 | 1,905 | 14 | 37 | 71,750 | 1,901 | |
| Total length | 735,000 | 17,162,595 | 20,203,737 | 12,891,907 | 1,169,206 | 15,885 | 29,400 | 54,292,998 | 1,166,416 | |
| No. of long contigs (≥ 1 kbp) | 54 | 3,828 | 3,416 | 2,065 | 24 | 11 | 9 | 9,548 | 23 | |
| No. of ultra-long contigs (≥ 50 kbp) | 0 | 0 | 0 | 0 | 0 | 0 | 0 | 0 | 0 | |
| Largest contig | 3,546 | 10,253 | 7,753 | 8,247 | 1,995 | 1,646 | 1,480 | 9,671 | 1,995 | |
| *N50* | 599 | 776 | 697 | 683 | 594 | 1,084 | 828 | 723 | 594 | |
| *L50* | 472 | 7,313 | 10,085 | 6,622 | 819 | 7 | 14 | 25,689 | 817 | |
|  | | | | | | | | | | |
|  | **Iowa** | | | | | | | | | |
|  | **CLC** | **IDBA-UD** | **MEGAHIT** | **metaSPAdes** | **MetaVelvet** | **Omega** | **Ray Meta** | **SPAdes** | **Velvet** | |
| Number of contigs (≥ 500 bp) | 408,385 | N/A | 984,964 | 727,092 | 5,211 | 47 | 36,990 | N/A | 2,627 | |
| Total length | 432,703,575 | N/A | 1,036,490,541 | 873,829,053 | 3,997,155 | 114,251 | 44,856,058 | N/A | 1,860,815 | |
| No. of long contigs (≥ 1 kbp) | 114,196 | N/A | 277,623 | 225,046 | 727 | 31 | 14,774 | N/A | 228 | |
| No. of ultra-long contigs (≥ 50 kbp) | 4 | N/A | 11 | 100 | 0 | 0 | 0 | N/A | 0 | |
| Largest contig | 70,207 | N/A | 104,841 | 188,499 | 15,640 | 15,697 | 48,390 | N/A | 15,640 | |
| *N50* | 1,107 | N/A | 1,096 | 1,339 | 741 | 5,820 | 1,389 | N/A | 663 | |
| *L50* | 95,222 | N/A | 235,226 | 138,932 | 1,842 | 7 | 8,781 | N/A | 973 | |

* N/A – Assembly could not be performed with these tools under the available resources.

**Table S4. Assembly statistics for the assembled aquatic metagenomes.**

|  | **Kolkata Lake** | | | | | | | | | | |
| --- | --- | --- | --- | --- | --- | --- | --- | --- | --- | --- | --- |
|  | **CLC** | **IDBA-UD** | **MEGAHIT** | **metaSPAdes** | **MetaVelvet** | **Omega** | **Ray Meta** | **SPAdes** | **Velvet** |  |  |
| Number of contigs (≥ 500 bp) | 181,128 | 221,734 | 324,600 | 5,927 | 147,420 | 28,201 | 19,945 | 6,388 | 138,176 |  |  |
| Total length | 176,334,760 | 239,616,234 | 330,771,089 | 5,695,829 | 133,768,764 | 51,164,188 | 32,776,885 | 6,378,576 | 106,649,169 |  |  |
| No. of long contigs (≥ 1 kbp) | 42,183 | 68,266 | 89,812 | 1,517 | 34,891 | 23,966 | 9,408 | 1,808 | 20,287 |  |  |
| No. of ultra-long contigs (≥ 50 kbp) | 2 | 2 | 4 | 1 | 0 | 0 | 0 | 0 | 0 |  |  |
| Largest contig | 58,518 | 67,794 | 81,287 | 71,858 | 37,935 | 49,562 | 49,052 | 47,837 | 12,417 |  |  |
| *N50* | 961 | 1,144 | 1,049 | 965 | 900 | 1,924 | 2,347 | 1,028 | 737 |  |  |
| *L50* | 45,488 | 53,482 | 81,952 | 1,618 | 43,665 | 6,179 | 3,279 | 1,696 | 47,957 |  |  |
|  | | | | | | | | | | | |
|  | **Tara Ocean** | | | | | | | | | | |
|  | **CLC** | **IDBA-UD** | **MEGAHIT** | **metaSPAdes** | **MetaVelvet** | **Omega** | **Ray Meta** | **SPAdes** | **Velvet** | |  |
| Number of contigs (≥ 500 bp) | 50,716 | 163,815 | 216,938 | 185,419 | 67,161 | 15,982 | 6,128 | 220,178 | 57,816 | |  |
| Total length | 46,069,409 | 179,686,756 | 210,621,485 | 202,770,058 | 55,972,515 | 34,861,819 | 7,277,214 | 275,920,632 | 45,425,460 | |  |
| No. of long contigs (≥ 1 kbp) | 10,720 | 50,498 | 56,243 | 48,640 | 12,590 | 13,305 | 2,179 | 70,711 | 8,802 | |  |
| No. of ultra-long contigs (≥ 50 kbp) | 0 | 2 | 1 | 37 | 0 | 9 | 0 | 54 | 0 | |  |
| Largest contig | 39,748 | 101,400 | 62,649 | 141,519 | 30,177 | 102,255 | 41,443 | 197,381 | 21,980 | |  |
| *N50* | 880 | 1,166 | 982 | 1,124 | 805 | 2,691 | 1,329 | 1,415 | 749 | |  |
| *L50* | 14,113 | 38,236 | 58,246 | 39,033 | 21,544 | 2,737 | 1,345 | 39,617 | 19,631 | |  |
|  | | | | | | | | | | | |
|  | **Arctic Frost Flower** | | | | | | | | | | |
|  | **CLC** | **IDBA-UD** | **MEGAHIT** | **metaSPAdes** | **MetaVelvet** | **Omega** | **Ray Meta** | **SPAdes** | **Velvet** | |  |
| Number of contigs (≥ 500 bp) | 3,645 | 10,024 | 7835 | 5,750 | 4,840 | 71 | 3,906 | 14,058 | 4,147 | |  |
| Total length | 6,214,696 | 12,554,320 | 10,702,435 | 9,367,300 | 5,297,757 | 57,772 | 3,985,954 | 16,431,291 | 3,260,116 | |  |
| No. of long contigs (≥ 1 kbp) | 2,112 | 1,650 | 1172 | 1,238 | 1,980 | 22 | 1,532 | 2,251 | 731 | |  |
| No. of ultra-long contigs (≥ 50 kbp) | 0 | 0 | 0 | 5 | 0 | 0 | 0 | 15 | 0 | |  |
| Largest contig | 35,081 | 37,705 | 48,918 | 73,164 | 6,410 | 2,960 | 6,355 | 142,838 | 3,321 | |  |
| *N50* | 2,236 | 1,551 | 4,142 | 5,113 | 1,241 | 1,010 | 1,091 | 1,137 | 784 | |  |
| *L50* | 758 | 896 | 500 | 407 | 1,373 | 22 | 1,256 | 1,670 | 1,479 | |  |

**Table S5. Assembly statistics for the assembled human gut metagenomes.**

|  | **Infant Gut** | | | | | | | | | |
| --- | --- | --- | --- | --- | --- | --- | --- | --- | --- | --- |
|  | **CLC** | **IDBA-UD** | **MEGAHIT** | **metaSPAdes** | **MetaVelvet** | **Omega** | **Ray Meta** | **SPAdes** | **Velvet** | |
| Number of contigs (≥ 500 bp) | 78,374 | 74,480 | 82,679 | 69,064 | 32,616 | 2 | 22,985 | 66,893 | 74,601 | |
| Total length | 155,498,363 | 168,421,610 | 195,409,115 | 199,621,178 | 88,068,229 | 1,988 | 57,640,536 | 203,097,710 | 114,330,088 | |
| No. of long contigs (≥ 1 kbp) | 37,980 | 37,139 | 37,983 | 35,394 | 20,258 | 1 | 13,845 | 34,196 | 30,098 | |
| No. of ultra-long contigs (≥ 50 kbp) | 110 | 117 | 273 | 333 | 33 | 0 | 77 | 400 | 12 | |
| Largest contig | 207,124 | 147,767 | 286,478 | 250,614 | 105,275 | 1,145 | 165,582 | 476,171 | 71,403 | |
| *N50* | 3,250 | 4,576 | 5,779 | 8,543 | 5,237 | 1,145 | 4,286 | 9,698 | 2,093 | |
| *L50* | 8,657 | 7,115 | 5,858 | 4,327 | 3,694 | 1 | 2,236 | 3,839 | 10,967 | |
|  | | | | | | | | | |  |
|  | **European Gut** | | | | | | | | | |
|  | **CLC** | **IDBA-UD** | **MEGAHIT** | **metaSPAdes** | **MetaVelvet** | **Omega** | **Ray Meta** | **SPAdes** | **Velvet** | |
| Number of contigs (≥ 500 bp) | 53,031 | 58,129 | 64,422 | 62,668 | 33,649 | 2,285 | 11,814 | 67,067 | 61,429 | |
| Total length | 89,451,760 | 132,103,940 | 87,777,313 | 140,509,146 | 78,332,430 | 3,363,693 | 21,606,086 | 149,369,596 | 87,676,277 | |
| No. of long contigs (≥ 1 kbp) | 21,434 | 26,451 | 24,709 | 24,355 | 19,494 | 1,971 | 5,123 | 26,175 | 24,838 | |
| No. of ultra-long contigs (≥ 50 kbp) | 63 | 132 | 13 | 241 | 53 | 0 | 8 | 247 | 1 | |
| Largest contig | 335,572 | 159,241 | 106,293 | 781,723 | 332,038 | 23,809 | 84,884 | 454,757 | 58,034 | |
| *N50* | 2,571 | 5,166 | 1,651 | 6,273 | 3,980 | 1,416 | 3,045 | 6,459 | 1,819 | |
| *L50* | 6,176 | 4,836 | 11,487 | 3,532 | 3,795 | 769 | 1,267 | 3,811 | 10,640 | |
|  | | | | | | | | | |  |
|  | **Scandinavian Gut** | | | | | | | | | |
|  | **CLC** | **IDBA-UD** | **MEGAHIT** | **metaSPAdes** | **MetaVelvet** | **Omega** | **Ray Meta** | **SPAdes** | **Velvet** | |
| Number of contigs (≥ 500 bp) | 54,768 | 75,394 | 82,546 | 77,794 | 59,108 | 14,936 | 8,567 | 79,226 | 58,981 | |
| Total length | 84,303,852 | 130,334,592 | 144,527,440 | 147,859,250 | 75,699,080 | 36,770,655 | 15,764,885 | 153,777,699 | 73,276,966 | |
| No. of long contigs (≥ 1 kbp) | 20,244 | 31,372 | 32,317 | 30,450 | 20,361 | 13,943 | 4,567 | 32,194 | 20,168 | |
| No. of ultra-long contigs (≥ 50 kbp) | 90 | 75 | 140 | 217 | 7 | 12 | 2 | 202 | 0 | |
| Largest contig | 216,892 | 159,248 | 313,634 | 268,823 | 66,119 | 81,480 | 83,243 | 317,289 | 48,882 | |
| *N50* | 2,113 | 2,720 | 2,897 | 3,615 | 1,496 | 2,900 | 2,784 | 3,720 | 1,434 | |
| *L50* | 6,877 | 8,744 | 8,115 | 5,998 | 10,785 | 2,847 | 1,428 | 6,356 | 11,425 | |

**Table S6. Assembly statistics for the assembled synthetic metagenomes.**

|  | **Low Complexity Synthetic Metagenome** | | | | | | | | |
| --- | --- | --- | --- | --- | --- | --- | --- | --- | --- |
|  | **CLC** | **IDBA-UD** | **MEGAHIT** | **metaSPAdes** | **MetaVelvet** | **Omega** | **Ray Meta** | **SPAdes** | **Velvet** |
| Number of contigs (>= 500 bp) | 22,03 | 25,37 | 34,397 | 17,262 | 18,693 | 514,919 | 10,221 | 15,426 | 24,719 |
| Total length | 82,906,659 | 84,760,377 | 85,371,336 | 94,237,845 | 17,976,720 | 319,705,390 | 58,171,311 | 99,869,764 | 23,035,683 |
| Number of contigs (>= 1000 bp) | 11,732 | 13,129 | 16,071 | 6,935 | 5,837 | 4,924 | 8,560 | 7,211 | 19,086 |
| Total bases (in contigs >= 1000 bp) | 75,346,850 | 76,305,826 | 73,014,567 | 87,195,256 | 9,172,593 | 22,330,416 | 54,472,631 | 95,004,922 | 11,073,683 |
| Largest contig | 1,363,103 | 601,708 | 475,109 | 1,362,249 | 6,241 | 5,027 | 960,879 | 1,299,913 | 6,241 |
| *N50* | 31,952 | 11,06 | 6,283 | 62,213 | 1,016 | 622 | 74,091 | 64,229 | 970 |
| *L50* | 309 | 1,387 | 2,348 | 283 | 5,655 | 210,063 | 119 | 280 | 7,663 |
| Genome Fraction | 64.919 | 64.98 | 60.903 | 69.883 | 11.492 | 48.674 | 44.475 | 71.244 | 14.703 |
| Number of Misassemblies | 241 | 1,314 | 360 | 272 | 8 | 674 | 227 | 643 | 10 |
| Unaligned length | 589,665 | 141,354 | 86,957 | 24,456 | 541 | 179,313 | 2,734 | 46,489 | 604 |
| Number of N's per 100 kbp | 286.22 | 0 | 0 | 30.92 | 0 | 0 | 209.7 | 119.96 | 0 |
| Number of mismatches per 100 kbp | 183.59 | 116.87 | 310.63 | 211.39 | 150.44 | 1,804.9 | 155.05 | 221.71 | 131.95 |
| Number of indels per 100 kbp | 9.87 | 0.83 | 0.9 | 1.63 | 0.23 | 2.51 | 1.33 | 1.49 | 0.19 |

|  | **Medium Complexity Synthetic Metagenome** | | | | | | | | | |
| --- | --- | --- | --- | --- | --- | --- | --- | --- | --- | --- |
|  | **CLC** | **IDBA-UD** | **MEGAHIT** | **metaSPAdes** | **MetaVelvet** | **Omega** | **Ray Meta** | **SPAdes** | **Velvet** |  |
| Number of contigs (>= 500 bp) | 80,528 | 84,037 | 119,591 | 71,191 | 77,767 | 209,183 | 7,771 | 64,224 | 77,663 |  |
| Total length | 185,168,654 | 222,289,879 | 257,341,686 | 238,619,539 | 68,674,229 | 141,462,232 | 85,654,354 | 262,581,596 | 68,600,419 |  |
| Number of contigs (>= 1000 bp) | 38,962 | 40,724 | 52,606 | 30,280 | 19,205 | 21,662 | 5,368 | 30,840 | 19,195 |  |
| Total bases (in contigs >= 1000 bp) | 154,074,665 | 192,559,057 | 211,591,582 | 210,833,358 | 29,287,685 | 26,259,657 | 84,075,855 | 239,673,252 | 29,276,578 |  |
| Largest contig | 1,122,639 | 286,805 | 352,739 | 1,046,380 | 9,610 | 5,188 | 974,677 | 1,626,065 | 9,610 |  |
| *N50* | 4,590 | 6,857 | 4,824 | 17,027 | 894 | 661 | 45,663 | 32,692 | 894 |  |
| *L50* | 4,628 | 6,114 | 9,068 | 2,354 | 24,555 | 81,165 | 286 | 1,316 | 24,516 |  |
| Genome Fraction | 41.529 | 51.511 | 57.678 | 49.997 | 15.016 | 13.729 | 19.508 | 51.722 | 15.007 |  |
| Number of Misassemblies | 445 | 3,362 | 1,468 | 729 | 33 | 376 | 289 | 4,928 | 33 |  |
| Unaligned length | 1,232,136 | 470,585 | 456,588 | 244,737 | 37,279 | 68,470 | 882 | 891,242 | 13,210 |  |
| Number of N's per 100 kbp | 157.11 | 0 | 0 | 41.76 | 0.81 | 0 | 122.19 | 16.04 | 0 |  |
| Number of mismatches per 100 kbp | 246.46 | 136.46 | 394.19 | 272.13 | 248.38 | 1,538.1 | 112.91 | 338.47 | 247.94 |  |
| Number of indels per 100 kbp | 11.98 | 2.47 | 2.42 | 3.34 | 0.8 | 2.65 | 1.94 | 4.07 | 0.8 |  |
|  |  |  |  |  |  |  |  |  |  |  |
|  | **High Complexity Synthetic Metagenome** | | | | | | | | | |
|  | **CLC** | **IDBA-UD** | **MEGAHIT** | **metaSPAdes** | **MetaVelvet** | **Omega** | **Ray Meta** | **SPAdes** | **Velvet** |  |
| Number of contigs (>= 500 bp) | 266,055 | 461,372 | 787,673 | 631,776 | 279,516 | 7,577 | 28,925 | 570,793 | 294,268 |  |
| Total length | 373,238,950 | 725,516,659 | 1,067,622,317 | 908,158,293 | 368,586,285 | 7,035,938 | 34,533,650 | 1,106,296,159 | 243,613,789 |  |
| Number of contigs (>= 1000 bp) | 99,638 | 180,549 | 281,359 | 210,180 | 99,149 | 4,336 | 8,391 | 256,527 | 59,437 |  |
| Total bases (in contigs >= 1000 bp) | 250,047,413 | 532,985,992 | 721,217,607 | 622,465,729 | 245,505,326 | 4,984,746 | 20,682,392 | 887,607,518 | 88,162,814 |  |
| Largest contig | 619,843 | 209,198 | 297,972 | 1,474,910 | 233,272 | 2,777 | 52,656 | 917,112 | 8,911 |  |
| *N50* | 1,523 | 2,204 | 1,613 | 1,850 | 1,553 | 1,057 | 1,279 | 3,266 | 815 |  |
| *L50* | 47,109 | 60,407 | 130,896 | 81,827 | 49,213 | 2,909 | 5,345 | 54,673 | 96,915 |  |
| Genome Fraction | 22.031 | 36.972 | 52.289 | 44.158 | 22.501 | 0.492 | 2.387 | 47.787 | 13.601 |  |
| Number of Misassemblies | 1,420 | 52,099 | 14,106 | 4,521 | 581 | 18 | 30 | 77,264 | 227 |  |
| Unaligned length | 4,717,919 | 8,695,557 | 8,236,381 | 4,583,659 | 558,470 | 12,659 | 961 | 19,523,496 | 230,175 |  |
| Number of N's per 100 kbp | 145.08 | 0 | 0 | 28.37 | 0.68 | 0 | 162.2 | 0.95 | 0 |  |
| Number of mismatches per 100 kbp | 619.25 | 402.97 | 811.83 | 665.82 | 418.32 | 1,509.26 | 129.65 | 810.46 | 394.53 |  |
| Number of indels per 100 kbp | 20.93 | 8.07 | 5.08 | 5.93 | 2.75 | 3.23 | 1.81 | 10.55 | 1.62 |  |

**Figure S1. Nonpareil estimates of sequence coverage (redundancy) for the 3 synthetic metagenomes studied.**

**
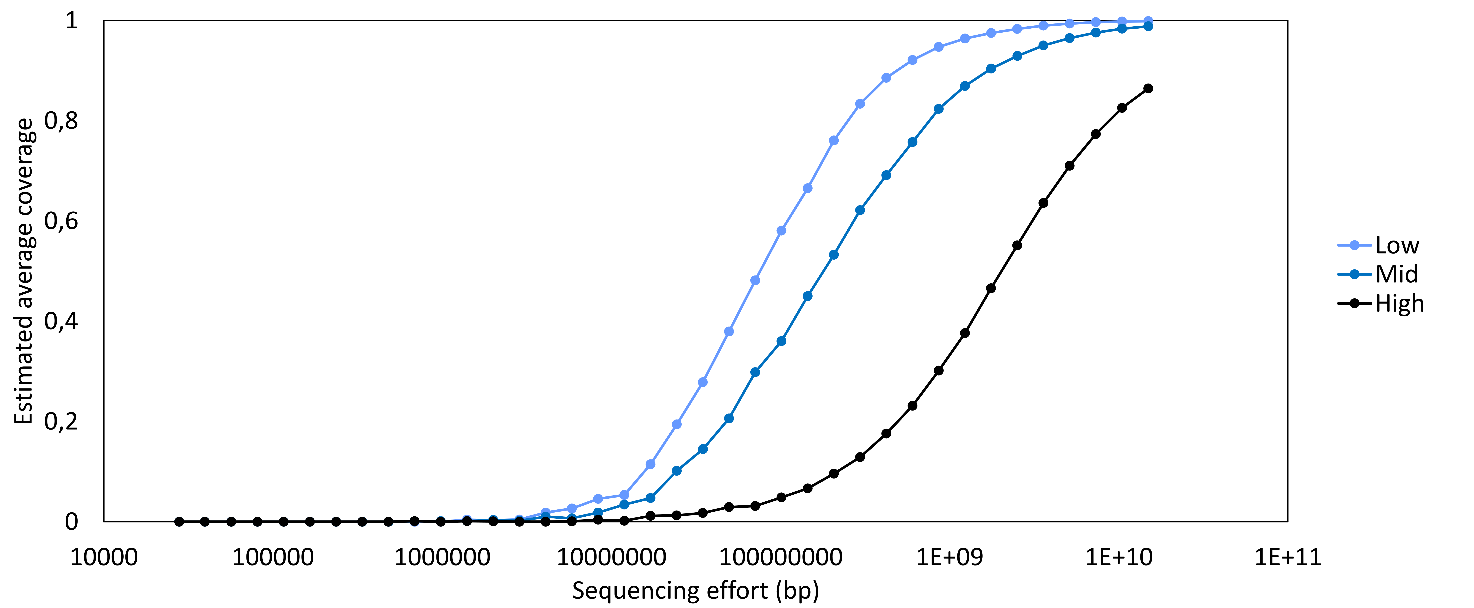
**

**Figure S2.** **Computational requirements of a Tara Ocean metagenome.** A) Total assembly span proportional to wall time required. B) Total assembly span in relation to peak memory usage.

**A**


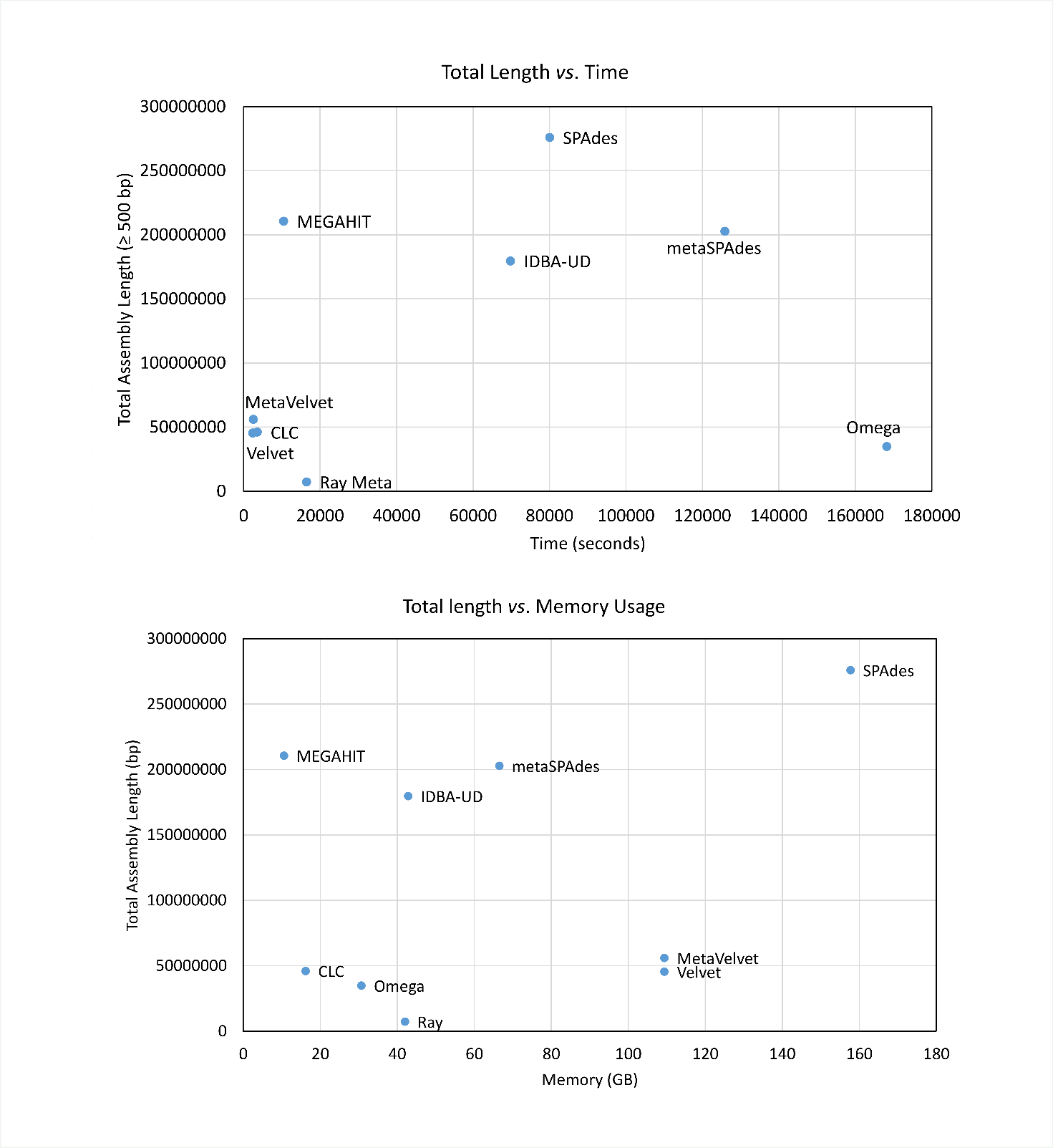


**B**

**Figure S3. Correlation between assembly span and mapping rate**. The exponential trendline indicates a very strong positive correlation between the amount of data utilized and the size of the generated assembly (R^2^ = 0.83).


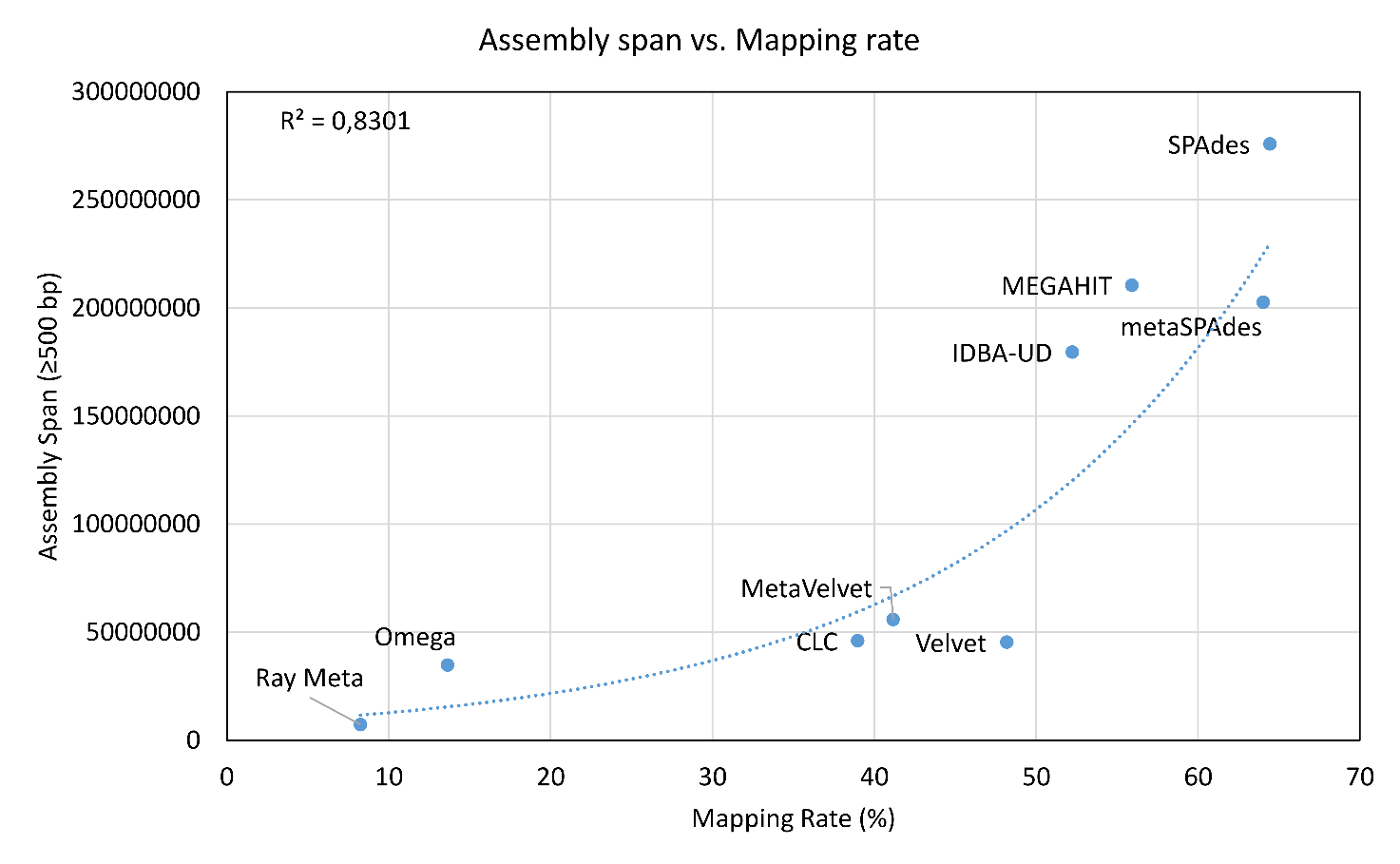


**References**

1 Peng, Y., Leung, H. C., Yiu, S.-M. & Chin, F. Y. IDBA-UD: a de novo assembler for single-cell and metagenomic sequencing data with highly uneven depth. *Bioinformatics* **28**, 1420-1428 (2012).

2 Li, D., Liu, C.-M., Luo, R., Sadakane, K. & Lam, T.-W. MEGAHIT: an ultra-fast single-node solution for large and complex metagenomics assembly via succinct de Bruijn graph. *Bioinformatics*, btv033 (2015).

3 Nurk, S., Meleshko, D., Korobeynikov, A. & Pevzner, P. metaSPAdes: a new versatile de novo metagenomics assembler. *arXiv preprint arXiv:1604.03071* (2016).

4 Namiki, T., Hachiya, T., Tanaka, H. & Sakakibara, Y. MetaVelvet: an extension of Velvet assembler to de novo metagenome assembly from short sequence reads. *Nucleic acids research* **40**, e155-e155 (2012).

5 Haider, B. *et al.* Omega: an Overlap-graph de novo Assembler for Metagenomics. *Bioinformatics*, btu395 (2014).

6 Boisvert, S., Raymond, F., Godzaridis, É., Laviolette, F. & Corbeil, J. Ray Meta: scalable de novo metagenome assembly and profiling. *Genome biology* **13**, 1 (2012).

7 Bankevich, A. *et al.* SPAdes: a new genome assembly algorithm and its applications to single-cell sequencing. *Journal of Computational Biology* **19**, 455-477 (2012).

8 Zerbino, D. R. & Birney, E. Velvet: algorithms for de novo short read assembly using de Bruijn graphs. *Genome research* **18**, 821-829 (2008).

9 Howe, A. C. *et al.* Tackling soil diversity with the assembly of large, complex metagenomes. *Proceedings of the National Academy of Sciences* **111**, 4904-4909 (2014).

10 Hultman, J. *et al.* Multi-omics of permafrost, active layer and thermokarst bog soil microbiomes. *Nature* (2015).

11 Luo, C. *et al.* Soil microbial community responses to a decade of warming as revealed by comparative metagenomics. *Applied and environmental microbiology* **80**, 1777-1786 (2014).

12 Sunagawa, S. *et al.* Structure and function of the global ocean microbiome. *Science* **348**, 1261359 (2015).

13 Bowman, J. S., Berthiaume, C. T., Armbrust, E. V. & Deming, J. W. The genetic potential for key biogeochemical processes in Arctic frost flowers and young sea ice revealed by metagenomic analysis. *FEMS microbiology ecology* **89**, 376-387 (2014).

14 Bäckhed, F. *et al.* Dynamics and stabilization of the human gut microbiome during the first year of life. *Cell host & microbe* **17**, 690-703 (2015).

15 Karlsson, F. H. *et al.* Gut metagenome in European women with normal, impaired and diabetic glucose control. *Nature* **498**, 99-103 (2013).

16 Tremaroli, V. *et al.* Roux-en-Y gastric bypass and vertical banded gastroplasty induce long-term changes on the human gut microbiome contributing to fat mass regulation. *Cell metabolism* **22**, 228-238 (2015).
